# MRI-derived periventricular diffusivity contextualizes plasma pTau217 associations with Alzheimer pathology: a discovery–validation study

**DOI:** 10.21203/rs.3.rs-10895317/v1

**Published:** 2026-09-10

**Authors:** Rui Bao, Shiqi Zhang, Fei Wang, Weibo Cheng, Tonghua Zhang, Wencai Ding

**Affiliations:** The Second Affiliated Hospital of Wannan Medical University; The Second Affiliated Hospital of Wannan Medical University; The Second Affiliated Hospital of Wannan Medical University; The Second Affiliated Hospital of Wannan Medical University; The Second Affiliated Hospital of Wannan Medical University; The Second Affiliated Hospital of Wannan Medical University

**Keywords:** Alzheimer’s disease, plasma pTau217, periventricular diffusivity, amyloid PET, tau PET

## Abstract

**Background:**

Plasma phosphorylated tau 217 (pTau217) is a promising scalable marker of Alzheimer pathology, but discordance with positron emission tomography (PET) limits context-free interpretation. We tested whether magnetic resonance imaging (MRI)-derived periventricular diffusivity (PVeD) modifies associations between pTau217 and Alzheimer pathology.

**Methods:**

In this observational discovery–validation study, standardized pTau217 × PVeD interactions were tested for amyloid PET, tau PET, structural and vascular MRI, and cognitive outcomes in 602 participants from the Alzheimer’s Disease Neuroimaging Initiative (ADNI) and 2,313 participants from the Health and Aging Brain Study–Health Disparities (HABS-HD). Analyses were adjusted for prespecified covariates and false discovery rate correction.

**Results:**

In ADNI, pTau217 associations with amyloid Centiloid (interaction β = −0.096, 95% confidence interval [CI] − 0.154 to − 0.037; adjusted P = .018) and meta-temporal tau PET (β = −0.092, 95% CI − 0.158 to − 0.027; adjusted P = .018) were stronger at lower PVeD. Among participants with high pTau217, low PVeD was associated with faster cognitive decline than high PVeD (difference = − 0.100 Preclinical Alzheimer Cognitive Composite [PACC] units/year, 95% CI − 0.161 to − 0.038; adjusted P = .002). HABS-HD replicated the amyloid interaction (β = −0.109, 95% CI − 0.147 to − 0.070; adjusted P < .001) and multiple tau PET interactions; the high-pTau217/low-PVeD profile also declined faster (difference = − 0.037 PACC units/year; adjusted P = .002).

**Conclusions:**

PVeD reproducibly contextualized associations between plasma pTau217 and PET-defined Alzheimer pathology across cohorts with different participant composition, pTau217 assays, PET tracers, and cognitive measures. The findings motivate prospective evaluation of MRI-informed interpretation of blood biomarkers, but PVeD is an indirect diffusion marker and the observational design does not establish mechanism, clinical utility, or decision thresholds.

## Background

Earlier and more scalable assessment of Alzheimer’s disease (AD) pathology is increasingly important for prevention and disease-modifying trials, and blood-based biomarkers are helping enable this transition [1–4]. Among them, plasma phosphorylated tau 217 (pTau217) has shown high accuracy for identifying amyloid and tau pathology and substantial potential for trial prescreening [5–7]. Nevertheless, pTau217 is not interchangeable with positron emission tomography (PET). Discordant pTau217–amyloid PET and pTau217–tau PET profiles occur across the disease continuum and are associated with different biomarker and cognitive trajectories [8]. Two-cutoff workflows consequently retain an intermediate group that requires confirmatory testing [9], and combined plasma pTau217 and tau PET models provide modest but measurable prognostic gains over either biomarker alone [10]. The translational challenge is therefore not whether pTau217 is informative, but how its relationship with brain pathology should be interpreted across biologically heterogeneous individuals.

Magnetic resonance imaging (MRI)-derived and cognitive measures can inform brain vulnerability and prognosis [11, 12], whereas pTau217 workflows have mainly added blood biomarkers, demographics, or apolipoprotein E (APOE) genotype [9, 13]. In cognitively unimpaired adults, pTau217 outperformed other plasma biomarkers for identifying amyloid PET positivity, while a multivariable combination yielded only modest incremental discrimination [13]. Such additive approaches do not address a complementary question: whether the brain microenvironment modifies the association between circulating pTau217 and PET-defined AD pathology. An effect modifier could help explain why similar pTau217 concentrations correspond to different cerebral amyloid or tau burdens and support contextual, rather than stand-alone, interpretation of the blood biomarker.

Perivascular pathways contribute to fluid and solute transport within the brain [14]. Diffusion MRI measures sensitive to perivascular or periventricular water mobility may therefore capture a component of the tissue context in which AD pathology accumulates. Chen and colleagues recently introduced periventricular diffusivity (PVeD), an automated measure based on transverse diffusion within periventricular white matter adjacent to the lateral ventricles and encompassing the principal deep medullary veins [15]. Lower PVeD was associated with greater amyloid burden and adverse AD-related features, the inverse PVeD–amyloid association was reproducibly stronger in APOE ε4 carriers, and baseline PVeD predicted subsequent cognitive decline [15]. Related ADNI evidence using diffusion tensor imaging (DTI) analysis along the perivascular space (DTI-ALPS) linked lower values to subsequent amyloid accumulation, neurodegeneration, cognitive decline, and clinical progression [16]. Complementary Harvard Aging Brain Study evidence showed that cortical free water was associated with regional tau deposition, subsequent cognitive decline, and clinical progression, and interactions with amyloid or tau were associated with steeper cognitive decline [17]. PVeD is methodologically distinct from DTI-ALPS and measures water diffusion rather than bulk fluid transport; it should therefore be interpreted as an indirect, physiologically non-specific marker of the periventricular diffusion microenvironment, not as a direct measure of glymphatic flow or clearance. Whether PVeD modifies the association of plasma pTau217 with amyloid and tau pathology has not been established.

We hypothesised that associations of plasma pTau217 with PET-defined AD pathology would be stronger at lower PVeD. We tested this hypothesis in an observational discovery–validation study, using continuous pTau217 × PVeD interaction models in the Alzheimer’s Disease Neuroimaging Initiative (ADNI) and external validation in the Health and Aging Brain Study–Health Disparities (HABS-HD). The primary objective was to determine whether PVeD reproducibly modified pTau217 associations with amyloid and tau PET across cohorts that used different pTau217 assays and PET protocols. Secondary analyses examined MRI-derived neurodegeneration and vascular injury, cross-sectional cognition, and longitudinal cognitive decline to assess the scope and clinical relevance of the interaction. This design positioned PVeD as a candidate contextual marker rather than a replacement for pTau217 or PET. The biological rationale, imaging construct, and study framework are summarized in Fig. 1.

## Methods

### Study design and participants

We conducted an observational discovery–validation study using two independent ageing cohorts with multimodal AD biomarker data. ADNI served as the discovery cohort, and HABS-HD served as the external validation cohort [18, 19]. The primary analyses were cross-sectional and evaluated whether MRI-derived PVeD modified associations of plasma pTau217 with PET-defined AD pathology; MRI-derived neurodegeneration, vascular injury, and cognition were evaluated as complementary outcomes. Longitudinal cognitive analyses were secondary. All analyses were conducted separately by cohort, and participant-level data were not pooled.

### ADNI data source and governance

Data used in preparation of this article were obtained from the ADNI database (https://adni.loni.usc.edu/). ADNI was launched in 2003 as a public–private partnership led by Principal Investigator Michael W. Weiner, MD. Its original objective was to determine whether serial MRI, PET, other biological markers, and clinical and neuropsychological assessments could be combined to measure progression of mild cognitive impairment (MCI) and early AD. Its current objectives include biomarker validation for clinical trials, improved cohort diversity and generalisability, and provision of data on AD diagnosis and progression to the scientific community. Current information is available at https://adni.loni.usc.edu/.

ADNI participants were eligible when PVeD, plasma pTau217, age, sex, and education were available. The primary ADNI cohort comprised 602 participants; endpoint-specific sample sizes varied according to outcome and covariate availability and are reported with each analysis. HABS-HD participants were eligible for profile construction when PVeD and plasma pTau217 were available, yielding 2313 participants; complete-case samples for individual validation models were determined by the relevant outcome and covariates [18]. The parent studies obtained approval from their participating institutional review boards and written informed consent from participants or authorised representatives. This secondary analysis used deidentified data under the applicable data-use agreements.

### Diffusion MRI processing and PVeD calculation

PVeD values were generated using the automated workflow described by Chen and colleagues [15]. The PVeD algorithm is distinct from region-of-interest-based DTI-ALPS, although both approaches use axisspecific diffusion-tensor information; Huang and colleagues applied DTI-ALPS in ADNI as a related but methodologically separate measure [16]. Diffusion-weighted images were processed in DSI Studio (build dated March 18, 2023, as specified in the source PVeD workflow). Susceptibility distortion correction used Synthesized b0 for Diffusion Distortion Correction (Synb0-DISCO), which generated an undistorted b = 0 image from the T1-weighted image and paired it with the acquired distorted b = 0 image for TOPUP correction [20]. Eddy-current and motion correction was then performed with EDDY [21, 22]. Corrected images were reconstructed with q-space diffeomorphic reconstruction to International Consortium for Brain Mapping 152 (ICBM152) standard space [23]. The b-table orientation was verified against a population-averaged template, and diffusion-tensor measures were estimated from volumes with b values of 1000 s/mm^2^ or lower. A diffusion sampling length ratio of 1.25 and 2-mm isotropic output resolution were used. The resulting maps included mean diffusivity (MD) and axis-specific tensor components in the left–right, anterior–posterior, and inferior–superior directions (Dxx, Dyy, and Dzz, respectively).

The transverse tensor ratio (TTR) was calculated voxel-wise as $TTR=\frac{D_{xx}}{\sqrt{{D_{xx}}^{2}+{D_{yy}}^{2}+{D_{zz}}^{2}}},$ thereby expressing the transverse component relative to the three orthogonal tensor components. A white-matter mask was derived from the MD image. A predefined lateral-ventricle mask was registered to each participant’s MD image, and an anatomically constrained region-growing algorithm along the transverse axis generated the initial periventricular area, with maximal expansion around the ventricular body and progressively less expansion toward the ventricular margins. Intersection with the white-matter mask produced the final periventricular white-matter region. Left and right PVeD values were the median TTR within the corresponding regions; total PVeD, the prespecified primary measure, was the median TTR within the combined bilateral region and was not calculated as the arithmetic mean of the two hemispheric summaries [15]. PVeD was interpreted as an MRI-derived measure of the local periventricular diffusion microenvironment, not as a direct measurement of glymphatic flow.

Detailed diffusion-preprocessing parameters, quality-control criteria, and acquisition-sensitivity analyses are provided in the Supplementary Methods. Only values passing the source-pipeline quality-control procedures were retained. Total PVeD was used in primary models; quality indices were reviewed for technical adequacy and were not treated as biological covariates.

### Plasma biomarkers

ADNI plasma pTau217 was measured using the fully automated Lumipulse platform (Fujirebio Diagnostics); values designated invalid or unavailable because of insufficient sample volume in the released dataset were treated as missing [24]. In HABS-HD, the released Quanterix Simoa pTau217 measure was selected a priori as the primary validation biomarker because it provided the broadest available coverage; an additional pTau217 measure was retained for assay-specific sensitivity analyses [25]. Biomarker collection and assay procedures followed the protocols of the respective parent studies. Because concentrations were right-skewed, positive pTau217 values were log10-transformed and then standardised within cohort and assay before modelling; non-positive values were excluded from analyses requiring logarithmic transformation.

### PET, MRI, and cognitive outcomes

Primary pathology outcomes were amyloid and tau PET measures. ADNI amyloid burden was assessed using Centiloid and standardised uptake value ratio (SUVR) measures, and tau burden using meta-temporal and regional flortaucipir SUVR measures. The Centiloid scale was used as a standardised quantitative representation of amyloid PET burden [26]. HABS-HD validation outcomes included global florbetaben amyloid Centiloid and SUVR measures and [^18^F]PI-2620 medial temporal, posterior cingulate, lateral parietal, and mesial-temporal-lobe tau-stage-weighted SUVR measures derived with the cohort’s established processing pipeline [18]. MRI-derived complementary outcomes included hippocampal volume, cortical thickness, and white-matter-hyperintensity (WMH) burden where available. Imaging outcomes were analysed using processed measures released by the parent studies and were harmonised only at the construct and variable-definition levels; raw images and derived outcomes were not pooled across cohorts.

The primary ADNI cognitive endpoint was the Preclinical Alzheimer Cognitive Composite (PACC), specifically the ADNI-modified version with Trails B (mPACCtrailsB), obtained from the released ADNIMERGE dataset [27]. The ADNI modification combines z-standardised delayed word-list recall, Mini-Mental State Examination (MMSE), Logical Memory delayed recall, and Trail Making Test Part B scores, with higher values indicating better cognition [28]. HABS-HD validation used a harmonization-informed, cohort-specific PACC rather than an exact replication of the ADNI mPACCtrailsB. The primary HABS-HD PACC was the mean of internally standardised MMSE, Spanish and English Verbal Learning Test delayed recall, Digit Symbol Substitution, and animal fluency scores, and required at least three of four components; higher values indicated better cognition. Sensitivity definitions required all four components, substituted Trail Making Test Part B for animal fluency, or used neuropsychological components only.

### pTau217–PVeD profiles

For descriptive analyses, cohort-specific median cut points for pTau217 and PVeD defined four profiles: low pTau217/high PVeD, low pTau217/low PVeD, high pTau217/high PVeD, and high pTau217/low PVeD. These profiles were used for tables, figures, and descriptive longitudinal comparisons. Primary inference used continuous interaction models; median splits were not used to estimate the principal effect-modification parameter.

### Statistical analysis

Participant characteristics were summarised as mean (standard deviation [SD]), median (interquartile range [IQR]), or number (%), as appropriate. APOE ε4 status was coded as carrier versus non-carrier from cohort genotype data. For each continuous endpoint, ordinary least-squares linear regression included standardised log10 pTau217, standardised total PVeD, their product term, and prespecified covariates. ADNI primary models adjusted for age, sex, and education. Outcomes were standardised so that the pTau217 × PVeD coefficient represented the change in outcome SD units associated with a 1-SD-by-1-SD interaction. This interaction coefficient, with its 95% confidence interval (CI), was the prespecified estimand of effect modification; for adverse-burden outcomes, a negative coefficient indicated a stronger positive pTau217–outcome association at lower PVeD. Models involving MRI volume additionally accounted for intracranial volume when it was not already incorporated into the released measure.

The prespecified ADNI primary endpoint set comprised amyloid PET, tau PET, MRI-derived neurodegeneration, vascular injury, and cognition. To address multiplicity, interaction P values were adjusted with the Benjamini–Hochberg false discovery rate (FDR) procedure within each prespecified analysis set [29]. All tests were two-sided; P < 0.05 was considered nominally significant, and FDR-adjusted P < 0.05 was used to identify findings that remained supported after multiplicity correction. Prespecified sensitivity models additionally adjusted for APOE ε4 status, WMH burden, or hippocampal volume; restricted analyses to amyloid-positive participants; and adjusted for ADNI phase, DTI protocol description, or both. Incremental explanatory value was assessed with adjusted R^2^, Akaike information criterion, and Bayesian information criterion across nested models; these comparisons were not interpreted as validation of clinical prediction.

ADNI longitudinal cognition was summarized using participant-specific annual PACC slopes estimated from all eligible observations among participants with at least two assessments. Slopes were compared across the four profiles using linear models adjusted for age, sex, and education; the prespecified contrast compared the high pTau217/low PVeD profile with the high pTau217/high PVeD profile. APOE ε4 adjustment and direct repeated-measures mixed-effects models were evaluated as sensitivity and model-form analyses (Supplementary Methods).

HABS-HD validation used the same continuous pTau217 × PVeD interaction framework for standardized PET and cognitive outcomes. Primary cross-sectional models adjusted for age, sex, and APOE ε4 status. The all-available sample was primary, and a ± 24-month window provided temporal-alignment sensitivity analyses. Longitudinal profile contrasts adjusted for age, sex, and education, with APOE ε4 evaluated separately. Assay, composite-definition, and GFAP/NfL specificity analyses are detailed in the Supplementary Methods.

### Missing data, multiplicity, and reproducibility

Each model used complete cases for its outcome, exposure, modifier, and specified covariates; no imputation was performed for primary interaction analyses. Endpoint-specific denominators are reported in the tables and figures. Analyses were performed in R version 4.4.1 and Python version 3.12.4. Detailed cohort construction, variable definitions, sensitivity models, longitudinal audits, and validation analyses are provided in the Supplementary Methods and Supplementary Results.

## Results

### Cohort construction established complementary discovery and validation samples

The ADNI discovery cohort comprised 602 participants with MRI-derived PVeD and plasma pTau217 measurements. Endpoint-specific samples were nearly complete for cross-sectional amyloid PET (n = 589), tau PET (n = 595), and baseline cognition (n = 601), whereas 323 participants contributed longitudinal PACC data (Supplementary Figure S1; Supplementary Table S1). Median splits yielded four profiles of comparable size (n = 126–175; Table 1), supporting both continuous interaction and descriptive profile analyses.

Disease burden was concentrated in the high pTau217/low PVeD profile rather than explained by isolated demographic differences. This profile was oldest and had the greatest amyloid and tau burden, poorest baseline PACC, and highest prevalence of MCI or AD dementia (125 of 171 [73.1%]; Table 1; Fig. 2A–D). APOE ε4 carriage was similar in the two high-pTau217 profiles (57.4% vs 56.5%); thus, their phenotypic separation was not accompanied by differential ε4 enrichment.

HABS-HD provided a larger and more diverse validation sample (n = 2313), including 1591 participants with amyloid PET, 1042–1065 with regional tau PET, 2310 with baseline PACC data, and 1454 with repeated cognitive assessments (Supplementary Figure S1). The high pTau217/low PVeD profile again showed the highest amyloid and mesial-temporal tau burden, lowest cognition, and greatest proportion with MCI or dementia (212 of 647 [32.8%]; Fig. 2E–H), reproducing the descriptive risk gradient observed in ADNI.

### Lower PVeD strengthened pTau217–PET associations in ADNI

The primary interaction analysis identified a consistent pattern: lower PVeD strengthened the positive association of pTau217 with PET-defined pathology. This interaction was evident for amyloid Centiloid (n = 589; β = −0.096; 95% CI, − 0.154 to − 0.037; FDR-adjusted P = 0.018) and amyloid SUVR, and extended to meta-temporal tau (n = 595; β = −0.092; 95% CI, − 0.158 to − 0.027; FDR-adjusted P = 0.018) and four additional temporal/parietal regions (Table 2; Fig. 3A–C). Effect estimates were similar across the significant tau regions (β = −0.082 to − 0.099), indicating a distributed rather than single-region interaction.

Entorhinal and posterior cingulate tau estimates were directionally consistent but did not survive FDR correction. No corresponding interaction was detected for hippocampal volume, WMH, baseline PACC, or baseline MMSE (all FDR-adjusted P ≥ 0.102). Thus, the cross-sectional effect was concentrated in molecular PET outcomes rather than generalized to structural, vascular, or baseline cognitive measures.

The interaction pattern was robust across complementary analyses. Estimates remained stable after separate adjustment for APOE ε4 status, WMH burden, or hippocampal volume and remained directionally consistent in the amyloid-positive subgroup (Supplementary Tables S2 and S3; Fig. 3D). Detailed estimates are reported in the Supplementary Results.

### Low PVeD identified faster cognitive decline in ADNI

Among 323 participants followed for a median of 2.99 years, cognitive decline was concentrated in the high pTau217/low PVeD profile (adjusted PACC slope, − 0.236 units/year; 95% CI, − 0.277 to − 0.194), compared with − 0.136 units/year in the high pTau217/high PVeD profile (Table 3; Fig. 4A; Supplementary Table S4). Both low-pTau217 profiles showed substantially slower decline.

The prespecified difference between the two high-pTau217 profiles was − 0.100 PACC units/year (95% CI, − 0.161 to − 0.038; FDR-adjusted P = 0.002) and was unchanged after APOE ε4 adjustment (FDR-adjusted P = 0.003; Fig. 4B).

Across the five ADNI PET outcomes, adding pTau217 × PVeD produced small adjusted R^2^ gains of 0.005–0.013; these increments quantify explanatory value rather than clinical prediction (Supplementary Table S5). Alternative PVeD thresholds, included-versus-excluded comparisons, and ADNI phase/protocol sensitivity analyses were likewise consistent with the primary pattern (Supplementary Tables S6, S7, and S8).

### HABS-HD reproduced pathology and cognitive interactions

HABS-HD independently reproduced the interaction for amyloid and tau PET. Lower PVeD strengthened pTau217 associations with florbetaben amyloid Centiloid and global SUVR (both n = 1591; β = −0.109; 95% CI, − 0.147 to − 0.070; FDR-adjusted P < 0.001) and with posterior cingulate, lateral parietal, and mesial-temporal tau measures (FDR-adjusted P ≤ 0.005; Fig. 3E–F). These findings persisted in the ± 24-month temporal-alignment sample (Fig. 3H; Supplementary Tables S9 and S10).

Specificity analyses were less conclusive because biomarker overlap reduced samples to 62–168 participants. GFAP × PVeD and NfL × PVeD models therefore did not establish strict biomarker specificity (Supplementary Table S11).

The cognitive findings were directionally consistent with the PET results. The pTau217 × PVeD interaction was associated with the primary PACC endpoint in both all-available and ± 24-month samples (β = 0.092 and 0.090, respectively; both FDR-adjusted P < 0.001; Supplementary Table S12), with comparable estimates across alternative composite definitions. Longitudinally, the high pTau217/low PVeD profile declined 0.037 PACC units/year faster than the high pTau217/high PVeD profile (95% CI, − 0.061 to − 0.014; FDR-adjusted P = 0.002), and the estimate was essentially unchanged after additional adjustment for APOE ε4 status (difference, − 0.037; 95% CI, − 0.061 to − 0.014; FDR-adjusted P = 0.002; Fig. 4D–F).

## Discussion

In this discovery–validation study, PVeD modified the association between plasma pTau217 and PET-defined AD pathology. Lower PVeD was associated with stronger pTau217–amyloid and pTau217–tau PET relationships in ADNI, and the same interaction direction was reproduced for florbetaben amyloid PET and several [^18^F]PI-2620 tau PET outcomes in HABS-HD, including temporally aligned analyses. The high pTau217/low PVeD profile also showed faster longitudinal cognitive decline in both cohorts. By contrast, comparable interactions were not detected for hippocampal volume, WMH, or baseline cognition, and the interaction produced only modest increases in explained variance. Taken together, these findings position PVeD as an imaging-derived context for interpreting plasma pTau217 rather than as a replacement for pTau217 or PET.

The findings address an important source of residual heterogeneity in blood-based assessment of AD. Plasma pTau217 has high diagnostic accuracy for amyloid-defined disease across cohorts and assay platforms [3–7, 30]. However, plasma pTau217 and PET are not biologically interchangeable. Plasma pTau217 can become abnormal before an entorhinal tau PET signal is detectable, and plasma-positive/tau-PET-negative profiles are particularly common early in the disease course [8, 31]. Moreover, biofluid p-tau is more closely related to early amyloid-associated processes, whereas tau PET reflects fibrillar regional tau burden and is more strongly associated with neurodegeneration and cognition [32]. The present interaction therefore does not imply that PVeD changes the concentration of pTau217. Rather, it suggests that a given plasma pTau217 level does not map uniformly onto PET burden and that part of this between-person variation is associated with the periventricular diffusion environment.

One plausible interpretation is that lower PVeD captures an altered periventricular milieu in which water mobility, extracellular geometry, tissue microstructure, vascular factors, or solute exchange differ in ways that co-vary with AD pathology. The original PVeD study associated lower PVeD with greater amyloid burden and poorer cognition, with reproducible APOE ε4 modification and longitudinal cognitive findings, while related DTI-ALPS work linked perivascular diffusion measures to amyloid deposition and clinical progression [15, 16]. In a cohort of veterans with post-traumatic stress disorder, lower DTI-ALPS covaried with higher cortical mean diffusivity in AD-vulnerable regions and lower cortical thickness, but not with cortical amyloid burden [33]. This interpretation is also compatible with broader evidence that brain fluid transport is shaped by interacting perivascular, interstitial, vascular, and anatomical processes [14]. Nevertheless, the current data do not identify a transport or clearance mechanism. Diffusion-derived measures remain sensitive to fiber architecture, free water, ventricular-adjacent anatomy, acquisition, and processing choices, and DTI-ALPS itself has not been validated as a direct measure of glymphatic flow in humans [34]. PVeD uses a different computational strategy and should likewise be described as a periventricular diffusion measure, not as a direct physiological assay of glymphatic function or solute clearance.

The distribution of effects across outcomes and cohorts helps define the boundary of the finding. The concentration of interactions in amyloid and tau PET is consistent with a role in contextualizing molecular pathology, but the null hippocampal, WMH, and cross-sectional cognitive results do not establish biological specificity. Contemporary criteria distinguish biomarker-defined disease from clinical staging [2], and cognition reflects reserve, education, co-pathology, and other influences in addition to amyloid and tau burden [35]. Related multimodal evidence likewise indicates that amyloid burden can strengthen imaging-defined neurodegeneration–cognition pathways in preclinical AD and that perivascular diffusion measures may relate to plasma neuroaxonal injury and cortical microstructure across neurodegenerative conditions [36, 37]. Across frontotemporal lobar degeneration subtypes, cortical and subcortical diffusion abnormalities extended beyond regions of cortical thinning and were associated with disease severity and cognitive–language impairment, further supporting the sensitivity of diffusion-derived measures to tissue injury not fully captured by conventional macrostructural MRI [38]. These studies do not validate PVeD specifically, because cortical mean diffusivity, tract-based diffusion measures, cortical free water, and PVeD quantify distinct aspects of tissue microstructure; rather, they support interpreting diffusion-derived measures as contextual markers embedded within broader tissue-injury pathways. External validation in HABS-HD is therefore important because participant composition, plasma assay implementation, PET tracers, and regional definitions differed from ADNI. Amyloid PET provided the closest cross-cohort target and yielded the clearest replication in both all-available and 24-month-window analyses. Tau PET findings were directionally supportive but cannot be interpreted as exact tracer-by-tracer or region-by-region replication. In addition, GFAP × PVeD interactions for several HABS-HD tau outcomes and the small multimarker samples leave strict biomarker specificity unresolved.

The longitudinal cognitive findings extend the PET interaction toward clinical relevance. Plasma pTau217 and tau PET are established predictors of subsequent cognitive decline in cognitively unimpaired individuals [10]. In ADNI, the high pTau217/low PVeD profile declined 0.100 PACC units per year faster than the high pTau217/high PVeD profile; HABS-HD showed a concordant difference of 0.037 PACC units per year. Reproduction across non-identical cognitive composites supports the direction of the finding, but its interpretation remains more cautious than that of the PET results. The profiles were defined by cohort-specific medians, the high pTau217/low PVeD group already had a less favorable baseline clinical profile, and residual confounding by disease stage or reserve cannot be excluded. These analyses support further study of risk enrichment, not use of median-defined profiles as diagnostic thresholds or individual prognostic categories.

The potential clinical role of PVeD is therefore best framed as a second-layer context measure. The 2025 Alzheimer’s Association guideline supports blood-based biomarkers as triage tests or substitutes for amyloid PET or cerebrospinal fluid biomarkers only when assay-specific performance thresholds are met and within a comprehensive diagnostic evaluation [30]. Two-threshold pTau217 workflows similarly reserve confirmatory testing for uncertain results [9]. In this setting, an MRI-derived measure might eventually help characterize individuals with intermediate or discordant blood and imaging evidence. However, the observed adjusted R^2^ gains of 0.005–0.013 quantify explanatory improvement, not diagnostic accuracy, calibration, clinical utility, or reduced PET use. Establishing an intended clinical use would require prespecified prediction models, independent validation, assessment of discrimination and calibration, and decision-curve or cost-effectiveness analyses at clinically meaningful thresholds. The present study does not show that PVeD changes treatment eligibility, identifies treatment responders, or can replace confirmatory biomarkers.

Strengths of the study include a discovery–validation design across two deeply phenotyped ageing cohorts; integration of plasma biomarkers, diffusion MRI, amyloid PET, tau PET, structural and vascular MRI, and longitudinal cognition; and prioritization of continuous interaction models over dichotomized profiles. Cross-cohort amyloid consistency, directional tau validation, temporal-window analyses, protocol sensitivity analyses, and comparator outcomes reduced the likelihood that the principal observation arose from a single cohort, tracer, or outcome definition. Reporting null outcomes and exploratory GFAP and NfL analyses also made the limits of the proposed pTau217 specificity explicit. The inclusion of HABS-HD broadened racial and ethnic representation and tested transportability across cohorts with different participant composition, assays, PET tracers, and cognitive measures.

### Limitations

Several limitations remain. First, the observational design cannot determine whether lower PVeD contributes to stronger pTau217–PET coupling or whether the measures share upstream ageing, vascular, anatomical, or disease-related determinants. Second, PVeD is not a direct physiological measure, and full harmonization across scanners, vendors, diffusion protocols, and acquisition phases was unavailable despite reassuring protocol sensitivity analyses. Third, plasma, MRI, and PET were not uniformly concurrent; the HABS-HD 24-month analyses reduced but did not eliminate temporal misalignment. Fourth, differences in pTau217 assays, tau tracers, regional definitions, and cognitive composites limit exact cross-cohort comparability. Fifth, complete-case analyses may be affected by selection related to multimodal data availability, and the smaller GFAP and NfL subsets provided limited power for specificity testing. Finally, ADNI and HABS-HD are research cohorts and do not represent all population-screening or routine-care settings; cohort-specific median profiles should not be interpreted as clinical cut-points.

## Conclusions

PVeD reproducibly contextualized associations between plasma pTau217 and PET-defined Alzheimer pathology across two independent cohorts, with the strongest cross-cohort evidence for amyloid PET and supportive evidence for tau PET and longitudinal cognition. These findings support a multimodal interpretation framework in which a scalable blood biomarker is considered alongside MRI-derived brain context, rather than as a context-free result. Prospective, harmonized studies should determine the biological determinants and test–retest reproducibility of PVeD, and establish whether MRI-informed interpretation improves prediction, risk stratification, or clinical decisions beyond established blood and imaging biomarkers.

## Supplementary Material

This is a list of supplementary files associated with this preprint. Click to download.

- 03SupplementaryMaterialPVeDpTau217.docx

## Figures and Tables

**Figure 1:**
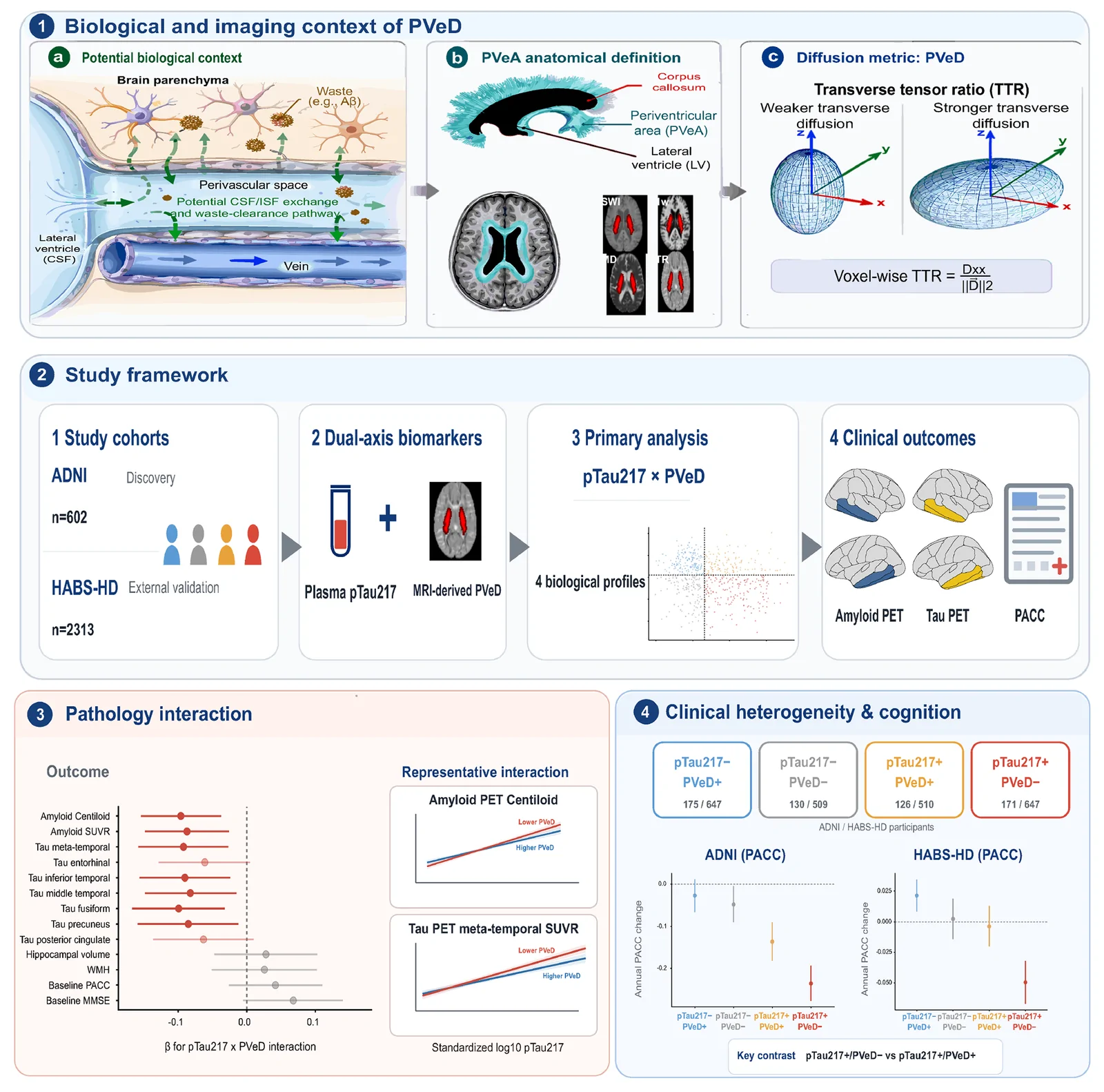
Biological and imaging context of PVeD and overview of the discovery–validation study. Section 1 illustrates the potential biological context of periventricular water mobility, the anatomical definition of the periventricular area, representative susceptibility-weighted, T1-weighted, mean-diffusivity, and transverse-tensor-ratio maps, and the tensor orientation underlying PVeD. The waste-clearance pathway is conceptual and does not establish a physiological clearance mechanism. Section 2 summarizes the ADNI discovery cohort and HABS-HD validation cohort, the dual-axis pTau217–PVeD framework, the individual-level ADNI pTau217–PVeD scatterplot with cohort-specific median-defined profiles, and PET and cognitive outcomes. Section 3 summarizes the pathology interaction evidence using standardized coefficients and representative model-predicted amyloid and tau outcomes at lower and higher PVeD. Section 4 shows profile sizes in both cohorts and the corresponding longitudinal cognitive patterns. Profile colors are used consistently: low pTau217/high PVeD, blue; low pTau217/low PVeD, gray; high pTau217/high PVeD, orange; and high pTau217/low PVeD, red.

**Figure 2:**
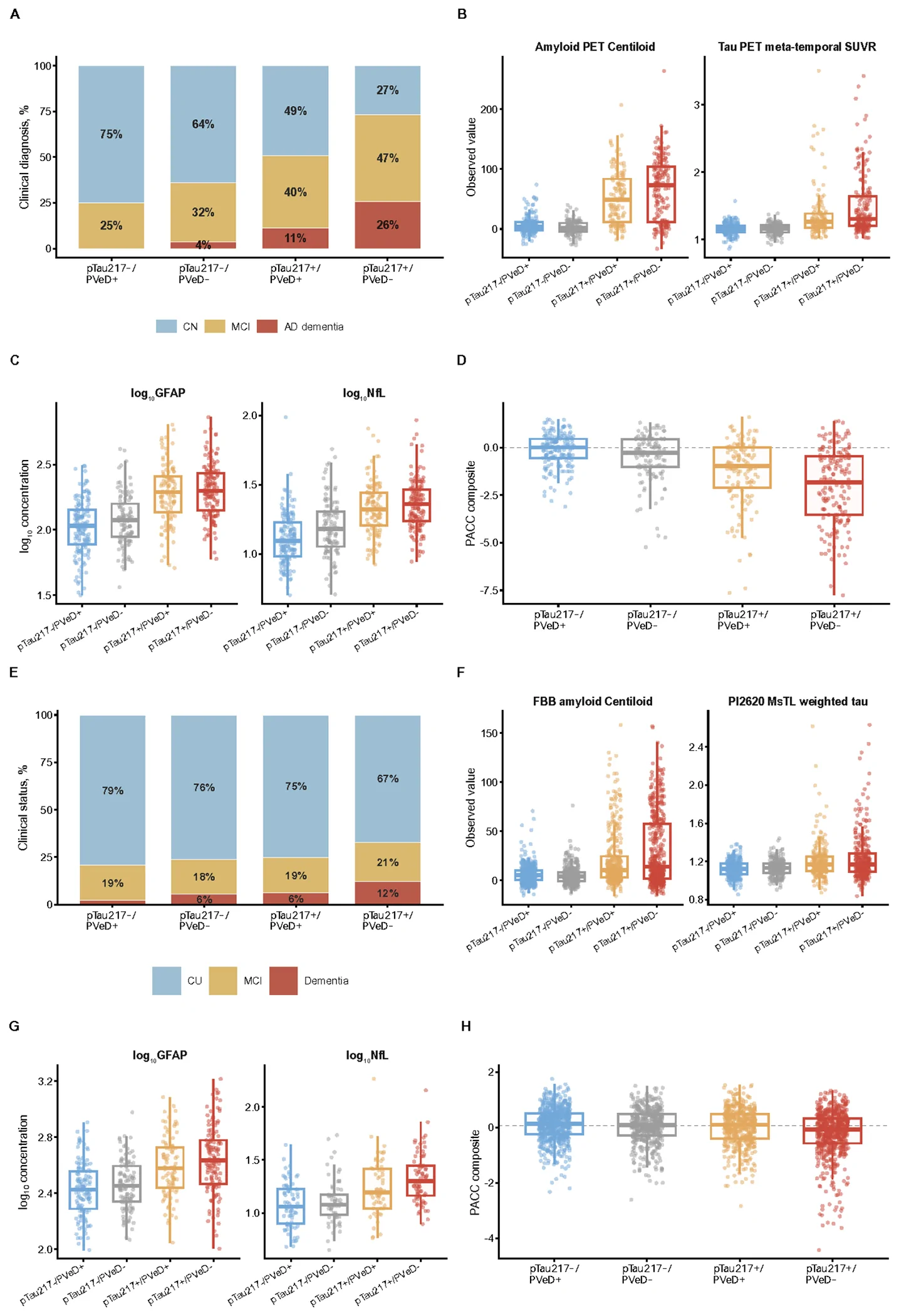
Profile-level clinical and biomarker phenotypes in ADNI and HABS-HD. Panels A–D show ADNI and panels E–H show HABS-HD. (A, E) Clinical composition of the four median-defined pTau217–PVeD profiles. (B, F) Participant-level distributions of amyloid PET burden and temporal tau PET burden. (C, G) Participant-level distributions of log10-transformed plasma GFAP and NfL. (D, H) Baseline PACC composite distributions. Points represent individual participants; boxes show the median and interquartile range, and whiskers extend to 1.5 times the interquartile range. Profile colors are used consistently: low pTau217/high PVeD, blue; low pTau217/low PVeD, gray; high pTau217/high PVeD, orange; and high pTau217/low PVeD, red. Clinical categories are CN, MCI, and AD dementia in ADNI and CU, MCI, and dementia in HABS-HD.

**Figure 3:**
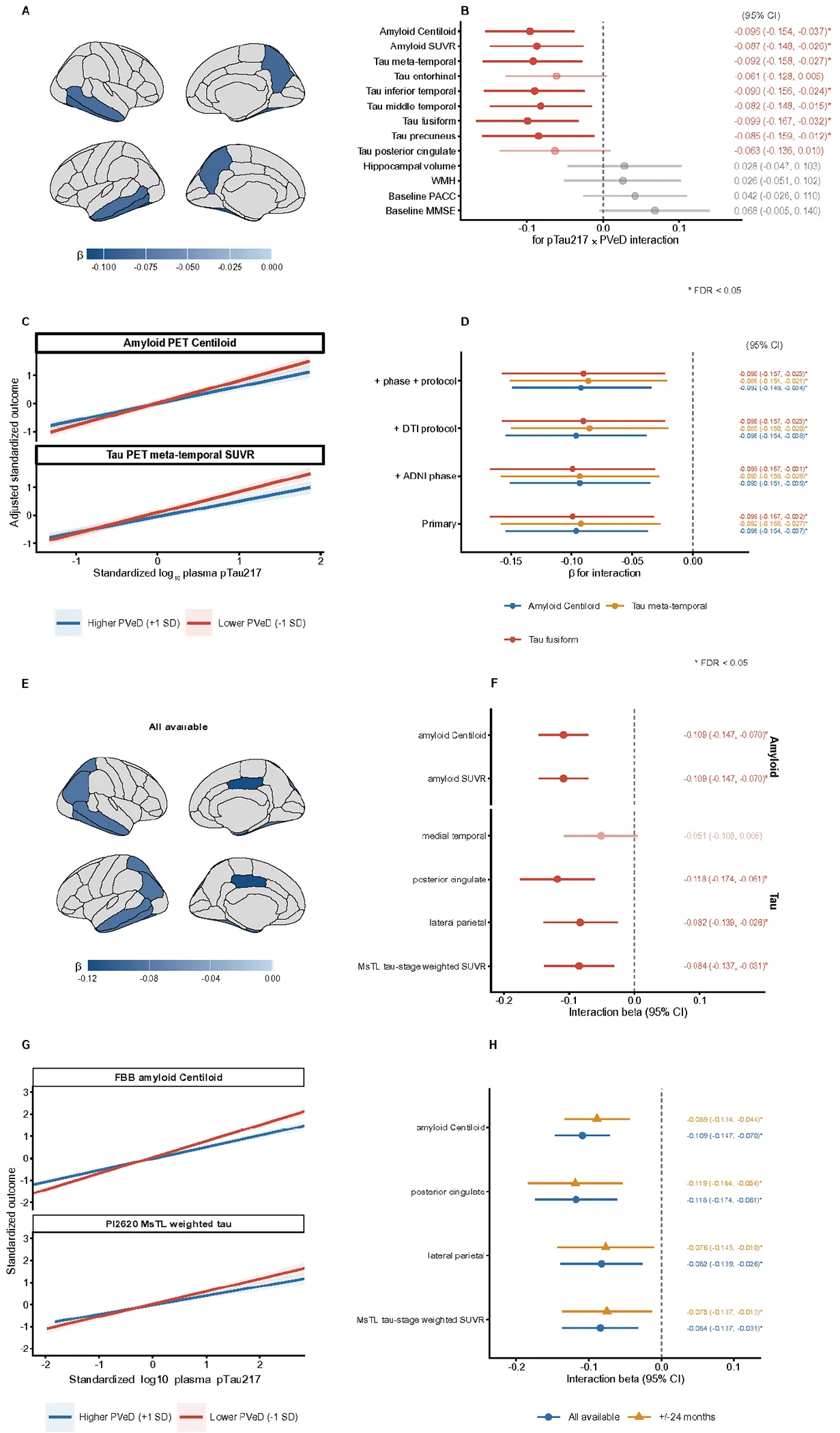
Continuous pTau217 × PVeD interactions with amyloid and tau PET in ADNI and HABS-HD. Panels A–D show ADNI and panels E–H show HABS-HD. (A, E) Cortical maps of standardized interaction coefficients for regional tau PET outcomes; blue regions indicate negative interactions supported after false discovery rate correction. (B, F) Standardized interaction coefficients and 95% confidence intervals for amyloid, tau, structural, vascular, and cognitive outcomes available in each cohort. Asterisks indicate false discovery rate-adjusted P < 0.05. (C, G) Model-predicted standardized amyloid and temporal tau outcomes across standardized log10 plasma pTau217 at lower PVeD (-1 standard deviation) and higher PVeD (+1 standard deviation); shaded bands indicate 95% confidence intervals. (D) ADNI sensitivity analyses after additional adjustment for phase, diffusion tensor imaging protocol, or both. (H) HABS-HD comparison of all-available and ±24-month time-aligned estimates. ADNI primary models were adjusted for age, sex, and education, and HABS-HD models for age, sex, and APOE ε4. Negative coefficients indicate a stronger positive association between pTau217 and PET pathology at lower PVeD.

**Figure 4:**
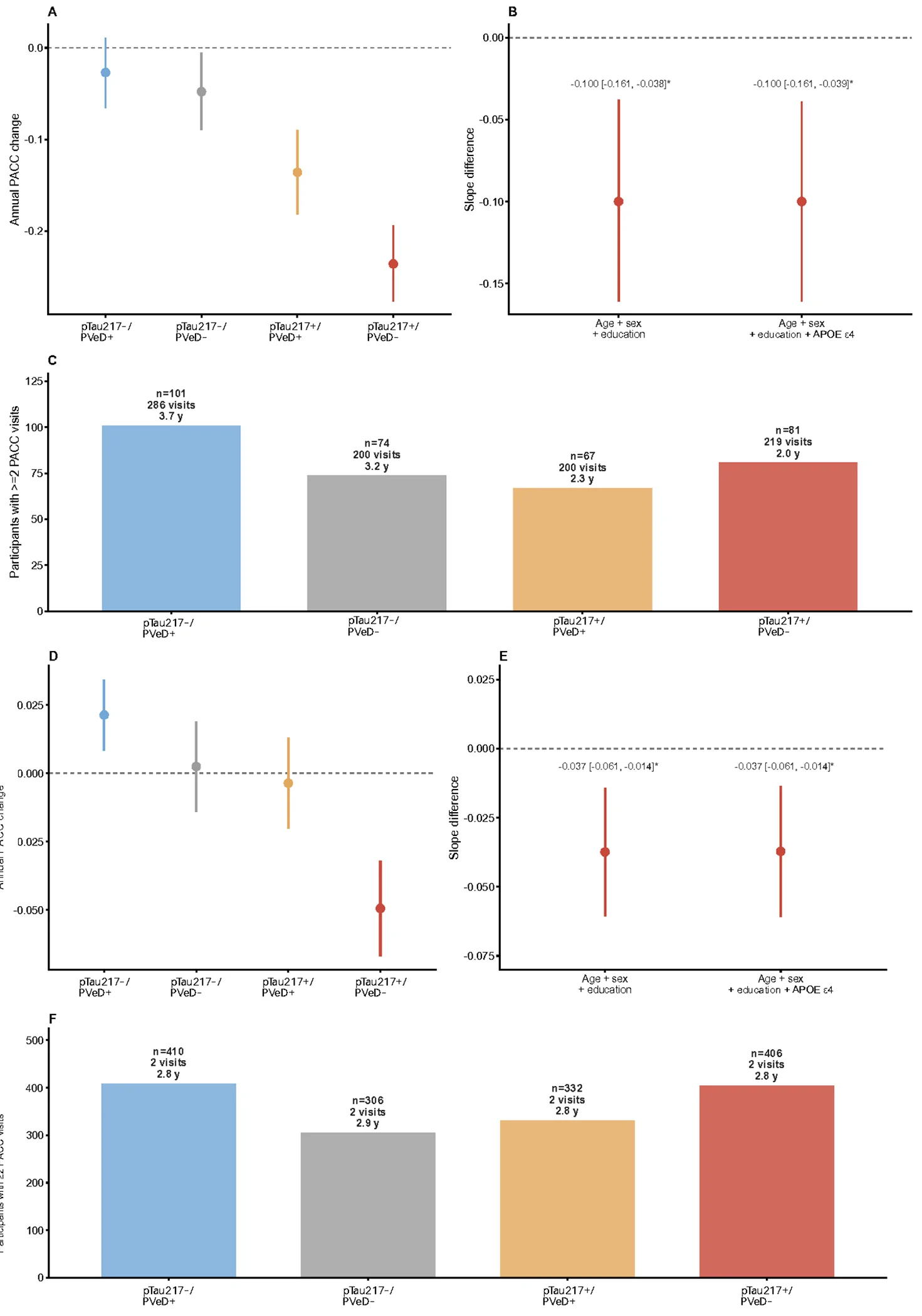
Longitudinal cognitive change across pTau217–PVeD profiles in ADNI and HABS-HD. Panels A–C show ADNI PACC and panels D–F show HABS-HD PACC results. (A, D) Adjusted annual cognitive change and 95% confidence intervals across the four median-defined profiles among participants with at least two assessments. Participant-specific annual slopes were estimated and then compared using linear models adjusted for age, sex, and education. (B, E) Prespecified annual-slope difference between the high pTau217/low PVeD and high pTau217/high PVeD profiles in the primary model and after additional adjustment for APOE ε4. Points indicate estimated differences, vertical lines indicate 95% confidence intervals, and numeric labels report estimates and 95% confidence intervals. Asterisks indicate false discovery rate-adjusted P < 0.05. (C, F) Longitudinal sample support by profile; annotations report participant numbers, total visits, and median follow-up in years. Profile colors are consistent with Figures 1 and 2.

**Table 1:** Selected baseline characteristics of the ADNI discovery cohort according to plasma pTau217–PVeD profiles.

Variable	pTau217− / PVeD+ (N = 175)	pTau217− / PVeD− (N = 130)	pTau217+ / PVeD+ (N = 126)	pTau217+ / PVeD− (N = 171)
Age, years	67.704 (6.877)	71.237 (7.821)	73.887 (7.597)	75.124 (7.284)
Education, years	16.297 (2.247)	16.208 (2.325)	15.921 (2.529)	16.456 (2.260)
Plasma pTau217	0.092 (0.028)	0.092 (0.029)	0.413 (0.351)	0.479 (0.323)
PVeD total	0.486 (0.019)	0.416 (0.032)	0.482 (0.019)	0.415 (0.031)
Amyloid PET Centiloid	7.608 (17.368)	2.935 (12.026)	52.854 (44.275)	66.873 (52.332)
Tau PET meta-temporal SUVR	1.162 (0.093)	1.154 (0.084)	1.396 (0.402)	1.513 (0.463)
Baseline PACC	−0.180 (0.977)	−0.546 (1.279)	−1.300 (1.705)	−2.020 (1.934)
Female sex, n (%)	128 (73.1%)	61 (46.9%)	81 (64.3%)	62 (36.3%)
APOE ε4 carrier, n/N (%)	50/170 (29.4%)	32/128 (25.0%)	70/122 (57.4%)	95/168 (56.5%)
MCI or AD dementia, n (%)	44 (25.1%)	47 (36.2%)	64 (50.8%)	125 (73.1%)

**Notes:** Continuous variables are presented as mean (SD), and categorical variables as n (%). Profiles were defined using cohort-specific medians of plasma pTau217 and PVeD. Low pTau217 / High PVeD, Low pTau217 / Low PVeD, High pTau217 / High PVeD, and High pTau217 / Low PVeD are denoted as pTau217− / PVeD+, pTau217− / PVeD−, pTau217+ / PVeD+, and pTau217+ / PVeD−, respectively. APOE ε4 carrier percentages use available APOE data (n=170, 128, 122, and 168, respectively). Clinical diagnosis is reported descriptively and was not included as a model covariate.

**Abbreviations:** AD, Alzheimer’s disease; ADNI, Alzheimer’s Disease Neuroimaging Initiative; APOE, apolipoprotein E; MCI, mild cognitive impairment; PACC, Preclinical Alzheimer Cognitive Composite; PET, positron emission tomography; pTau217, phosphorylated tau 217; PVeD, periventricular diffusivity; SD, standard deviation; SUVR, standardized uptake value ratio.

**Table 2:** Primary interaction analyses across PET, neuroimaging, and cognitive outcomes.

Domain	Outcome	N	pTau217 x PVeD beta (95% CI)	P value	FDR
Amyloid	Amyloid PET Centiloid	589	−0.096 (−0.154, −0.037)	0.001	0.018
Amyloid	Amyloid PET SUVR	589	−0.087 (−0.148, −0.026)	0.005	0.018
Tau	Tau PET meta-temporal SUVR	595	−0.092 (−0.158, −0.027)	0.005	0.018
Tau	Tau PET entorhinal SUVR	595	−0.061 (−0.128, 0.005)	0.071	0.102
Tau	Tau PET inferior temporal SUVR	578	−0.090 (−0.156, −0.024)	0.007	0.019
Tau	Tau PET middle temporal SUVR	578	−0.082 (−0.148, −0.015)	0.016	0.034
Tau	Tau PET fusiform SUVR	578	−0.099 (−0.167, −0.032)	0.004	0.018
Tau	Tau PET precuneus SUVR	578	−0.085 (−0.159, −0.012)	0.023	0.043
Tau	Tau PET posterior cingulate SUVR	578	−0.063 (−0.136, 0.010)	0.089	0.116
MRI neurodegeneration	Hippocampal volume	593	0.028 (−0.047, 0.103)	0.467	0.506
Vascular injury	White matter hyperintensity	593	0.026 (−0.051, 0.102)	0.513	0.513
Cognition	Baseline PACC	601	0.042 (−0.026, 0.110)	0.23	0.271
Cognition	Baseline MMSE	599	0.068 (−0.005, 0.140)	0.066	0.102

**Notes**: This table summarizes the primary pTau217 × PVeD interaction effects across amyloid PET, tau PET, structural MRI markers, and cognitive outcomes. Estimates are shown as beta coefficients with 95% confidence intervals. P values and false discovery rate (FDR)-adjusted P values are reported where applicable. The compact profile notation used throughout the manuscript is defined as follows: pTau217− / PVeD + = low pTau217 / high PVeD; pTau217− / PVeD− = low pTau217 / low PVeD; pTau217+ / PVeD + = high pTau217 / high PVeD; and pTau217+ / PVeD− = high pTau217 / low PVeD.

**Abbreviations:** CI, confidence interval; FDR, false discovery rate; MRI, magnetic resonance imaging; PET, positron emission tomography; pTau217, phosphorylated tau 217; PVeD, periventricular diffusivity; SUVR, standardized uptake value ratio; WMH, white matter hyperintensities.

**Table 3:** Longitudinal cognitive trajectories according to pTau217/PVeD profiles.

Outcome	N	Profile	Model	Estimate (95% CI)	P value	FDR
PACC composite	323	pTau217− / PVeD−	Age-, sex-, and education-adjusted model	−0.048 (−0.090, −0.005)	0.028	
PACC composite	323	pTau217+ / PVeD−	Age-, sex-, and education-adjusted model	−0.236 (−0.277, −0.194)	< 0.001	
PACC composite	323	pTau217− / PVeD+	Age-, sex-, and education-adjusted model	−0.027 (−0.066, 0.011)	0.164	
PACC composite	323	pTau217+ / PVeD+	Age-, sex-, and education-adjusted model	−0.136 (−0.182, −0.090)	< 0.001	
PACC composite	323	pTau217+ / PVeD− vs pTau217+ / PVeD+	Age-, sex-, and education-adjusted model	−0.100 (−0.161, −0.038)	0.002	0.002
PACC composite	323	pTau217+ / PVeD− vs pTau217+ / PVeD+	APOE ε4-adjusted sensitivity analysis	−0.100 (−0.161, −0.039)	0.001	0.003

**Notes**: This table presents longitudinal cognitive trajectories according to biomarker-defined pTau217/PVeD profiles. Estimates are reported with 95% confidence intervals and correspond to model-derived longitudinal change in cognitive performance. The profile notation was harmonized for consistency with the figures and manuscript text: pTau217− / PVeD+ denotes low pTau217 with high PVeD, pTau217− / PVeD− denotes low pTau217 with low PVeD, pTau217+ / PVeD+ denotes high pTau217 with high PVeD, and pTau217+ / PVeD− denotes high pTau217 with low PVeD.

**Abbreviations:** CI, confidence interval; FDR, false discovery rate; PACC, Preclinical Alzheimer Cognitive Composite; pTau217, phosphorylated tau 217; PVeD, periventricular diffusivity.

## Data Availability

The ADNI datasets analysed during the current study are available through the ADNI website (https://adni.loni.usc.edu/ ) upon registration and approval. The HABS-HD datasets are available through the HABS-HD website (https://healthandagingbrainstudy.org/ ) upon application and approval by the HABS-HD Data Access and Publication Committee. Scripts for the analytical methods and imaging processing are publicly available at https://github.com/dingneurology/PVeD-pTau217 and https://github.com/ChangleChen/EstPVeD.

## Abbreviations

AD: Alzheimer’s disease
ADNI: Alzheimer’s Disease Neuroimaging Initiative
HABS-HD: Health and Aging Brain Study–Health Disparities
MRI: magnetic resonance imaging
PACC: Preclinical Alzheimer Cognitive Composite
PET: positron emission tomography
pTau217: plasma phosphorylated tau 217
PVeD: periventricular diffusivity.
